# Retinal Pigment Epithelial Cells Mitigate the Effects of Complement Attack by Endocytosis of C5b-9

**DOI:** 10.4049/jimmunol.1500937

**Published:** 2015-08-31

**Authors:** Apostolos Georgiannakis, Tom Burgoyne, Katharina Lueck, Clare Futter, John Greenwood, Stephen E. Moss

**Affiliations:** Department of Cell Biology, University College London Institute of Ophthalmology, London EC1V9EL, United Kingdom

## Abstract

Retinal pigment epithelial (RPE) cell death is a hallmark of age-related macular degeneration. The alternative pathway of complement activation is strongly implicated in RPE cell dysfunction and loss in age-related macular degeneration; therefore, it is critical that RPE cells use molecular strategies to mitigate the potentially harmful effects of complement attack. We show that the terminal complement complex C5b-9 assembles rapidly on the basal surface of cultured primary porcine RPE cells but disappears over 48 h without any discernable adverse effects on the cells. However, in the presence of the dynamin inhibitor dynasore, C5b-9 was almost completely retained at the cell surface, suggesting that, under normal circumstances, it is eliminated via the endocytic pathway. In support of this idea, we observed that C5b-9 colocalizes with the early endosome marker EEA1 and that, in the presence of protease inhibitors, it can be detected in lysosomes. Preventing the endocytosis of C5b-9 by RPE cells led to structural defects in mitochondrial morphology consistent with cell stress. We conclude that RPE cells use the endocytic pathway to prevent the accumulation of C5b-9 on the cell surface and that processing and destruction of C5b-9 by this route are essential for RPE cell survival.

## Introduction

Age-related macular degeneration (AMD) is the leading cause of blindness in industrialized nations in people aged >65 y (1). In early AMD, the disease pathology typically affects the retinal pigment epithelial (RPE) cells and choriocapillaris, with the accumulation of extracellular lipoproteinaceous deposits (drusen) between the basal RPE cells and Bruch’s membrane (2). As the disease progresses, additional diffuse deposits form beneath the RPE cells that may contribute to cellular dysfunction by creating a barrier to diffusion between the RPE cells and the blood supply of the choroid (3, 4). Although the mechanisms that lead to the formation of these subretinal deposits are not understood, previous research demonstrated that they, and drusen, are rich in a number of inflammatory proteins, such as apolipoprotein E, amyloid P component, vitronectin, and complement proteins (e.g., C3b, C5, and C5b-9) (5–7). The accumulation of these deposits is suggestive of defects in complement regulation and is consistent with the presence of a number of genetic loci in complement genes associated with AMD susceptibility, in particular the single-nucleotide polymorphism in complement factor H (CFH) that switches Tyr^402^ to the risk-associated His^402^ (8–11). Additional risk alleles in the genes encoding C2, C3, C9, CFB, CFHR1, CFHR3, and CFI (12–16) point to a causative role for the innate immune system in AMD pathogenesis (17, 18).

Complement activation can be triggered by the classical, lectin, and alternative pathways and is normally kept in check by regulators, such as CFH. However, abnormalities in complement regulators and/or activators may lead to inappropriate activation of C3 and, ultimately, formation of the C5b-9 complex (19–22). C5b-9 assembly begins with the cleavage of C5 molecules into C5a and C5b via the C5 convertase (23). Then, C5b sequentially associates with the C6, C7, C8, and C9 complement proteins to assemble the membrane-associated C5b-7, C5b-8, and C5b-9 complexes (24). The number of C9 monomers that incorporates into the terminal complex is a determinant of the size of the C5b-9 pore; in bacteria and mammalian erythrocytes, the formation of multiple pores leads to death of the target cell (25, 26). However, nucleated cells are much more resistant to C5b-9, and rather than causing cell death, formation of the complex may stimulate cellular responses, such as a transient increase in intracellular calcium (27–29), activation of protein kinases (30), and changes in gene transcription (31). Of relevance to the pathogenesis of the neovascular form of AMD, sublytic C5b-9 was shown to increase the expression and secretion of vascular endothelial growth factor in RPE cells (27, 32, 33).

In the human retina, RPE cells form a critical interface in between the blood, circulating complement proteins, and the retina. Consequently, the basal aspect of the RPE cells is a site for C5b-9 assembly, and the complex was identified in the RPE cell/Bruch’s membrane in eyes as young as 5 y of age (34). The presence of C5b-9 increases with normal ageing, but it accumulates at higher levels in individuals with risk-associated AMD genotypes (35). In this study, we examined the mechanism used by RPE cells to eliminate C5b-9, because defects in this process may account for the accumulation of C5b-9 observed in AMD. We show that basal C5b-9 is rapidly cleared from the cell surface by endocytosis and that if this process is blocked, to mimic a dysfunctional clearance mechanism, the cells develop signs of mitochondrial stress, one of the hallmarks of the RPE cells in AMD (36). Although there is no direct evidence that the endocytic pathway is disrupted in AMD, our results suggest that, via its effects on mitochondria, chronic exposure to C5b-9 may contribute to RPE cell dysfunction, inflammasome activation (37), and the cellular pathology of AMD.

## Materials and Methods

### RPE cell isolation and culture

All experiments were performed using primary porcine RPE cells isolated from chilled porcine eyes freshly delivered from an abattoir. Once the eyes were detached from the surrounding muscle tissue, they were disinfected using PBS and Videne surgical scrub (Williams Medical Supplies; D748980) and placed at 4°C for 30 min in PBS and penicillin/streptomycin (1 mg/ml). The eyes were cut underneath the ora serrate, the anterior part (including the lens and vitreous) was discarded, and the posterior part (retina and RPE cells) was processed. The retina was detached from the RPE cell monolayer, cut at the optic nerve, and homogenized in KCl buffer (0.3 M KCl, 10 mM HEPES, 0.5 mM CaCl_2,_ and 1 mM MgCl_2_ [pH 7]) containing 48% sucrose solution. Trypsin-EDTA (10×; Life Technologies) was added to the posterior part of each eye (20 min at 37°C), and RPE cells were detached and isolated by repetitive pipetting. RPE cells were pelleted by centrifugation at 2000 rpm for 3 min, resuspended in fresh DMEM containing 10% FBS and 100 U/ml penicillin/streptomycin, and seeded into six-well plates (Nunclon Delta Surface; Thermo Scientific; 140675) at a density of ∼10^5^ cells/well. To make the basal surface of RPE cells accessible to the C5b-9 complex assembly, RPE cells were cultured on 12-well polyester Transwell inserts (catalog no. 734-1579; Corning). Cultivation of RPE cells in Transwells allowed cells to polarize and, therefore, mimic their morphology in the mammalian eye in vivo. Initially, cells were cultured in DMEM containing 10% FBS and 100 U/ml penicillin/streptomycin. Once they reached a confluent state, the amount of FBS was reduced to 1%. As a general practice, cells were maintained in Transwell inserts for 7–21 d prior to experimentation. To ensure the development of a properly formed monolayer within the Transwell plates, the transepithelial electrical resistance (TEER) was measured weekly, using an epithelial volt-ohm meter (World Precision Instruments), as well as after described treatments. To determine TEER (Ω/cm^2^), values of Transwells without cells were subtracted from the experimentally measured values. These values were then multiplied by the surface area of the Transwell.

### Formation of the C5b-9 complex

Cells were washed once with sterile PBS and incubated with 500 μl DMEM on the apical surface. On the basal surface, cells were treated with 500 μl DMEM containing the C5b-6 complex (0.2 mg/ml) and C7 (60 μg/ml), C8 (50 μg/ml), and C9 (60 μg/ml) purified complement proteins. Cells were incubated for 1, 4, 8, 24, and 48 h at 37°C/5% CO_2_, unless stated otherwise. As a control, C9 was omitted; thus, cells were treated with C5b-6 complex (0.2 mg/ml) and C7 (60 μg/ml) and C8 (50 μg/ml) purified complement proteins only. Purified complement component proteins referenced above were purchased from Complement Technology, unless stated otherwise. Normal human serum was purchased from Merck Millipore (S1-100ML), and heat inactivation was carried out at 56°C for 1 h.

### Inhibition of endocytosis using Dynasore

To block endocytosis of the C5b-9 complex, media was supplemented with 200 μg/ml Dynasore hydrate (Sigma; D7693) for 24 h (unless stated otherwise) at 37°C/5% CO_2_. The next day, cells were washed once with sterile PBS and fixed for immunofluorescence or electron microscopy.

### Immunofluorescence

Cells growing on culture well inserts were washed twice with sterile PBS and fixed with pure ice-cold methanol (5 min) or 4% paraformaldehyde (Sigma-Aldrich; 15,812-7) for 30 min at room temperature. Fixation was stopped by washing the cells thoroughly three times with PBS. Cells were permeabilized with PBS containing 0.01% Triton X-100 (PBS-T; Sigma; T-8787) for 15 min. To reduce background staining, the cells were blocked with 1% BSA (Sigma; A7906) in PBS-T for 1 h at room temperature. After blocking, the insert membrane was removed using a scalpel and placed on Parafilm prior to being incubated with a primary Ab (depending on the protein of interest). All primary Abs were diluted 1:50 in 1% BSA and PBS-T overnight at 4°C. The next day, cells were washed three times with PBS to remove unbound primary Ab. Then a secondary FITC- or TRITC-conjugated Ab was applied at 1:100 dilution for 1 h at room temperature. When examination of the cytoskeleton (F-actin) was required, rhodamine phalloidin (1:2000 dilution; Life Technologies; R415) was added together with the secondary Ab. To stain cell nuclei, DAPI (Sigma, U.K.) was added (1 mg/ml) 10 min before the removal of the secondary Ab. Excess secondary Ab was removed with three PBS washes. Cells were coated with mounting media (Mowiol), and a glass coverslip was placed on top. The membrane/coverslip were secured on a glass slide using mounting media for analysis using an inverted Leica SP2 confocal microscope. To acquire three-dimensional (3D) images of Z-sections, data were processed using Imaris 3D reconstruction software.

### Abs

The primary Abs used were Tim23 mouse monoclonal (BD Transduction Laboratories; 611222), C5b-9 mouse monoclonal (Dako Cytomation; M0777), EEA-1 rabbit polyclonal (Santa Cruz; L2211), and cathepsin D goat polyclonal IgG (R&D Systems; AF1029). CD55 and CD59 Abs were generously provided by Professor Paul Morgan (University of Cardiff, Cardiff, U.K.): clone MD1, a rat IgG1 mAb with species cross-reactivity against mouse, human, and pig, and clone 7A6, a mouse mAb with the same species cross-reactivity, respectively. The phospho-ERK Ab was mouse monoclonal E-4 from Santa Cruz (sc-7383), the ERK1/2 Ab was a rabbit polyclonal (Cell Signaling; 9102), and the heat shock protein 70-kDa chaperone was a mouse monoclonal (Santa Cruz; sc-7298).

### Electron microscopy and quantification of mitochondria

To assess whether the basal C5b-9 complex affects mitochondrial integrity (by measuring mitochondrial number), the following samples were used: nontreated RPE cells, cells incubated with DMEM + DMSO for 24 h (vehicle), cells incubated with DMEM containing 200 μg/ml dynasore hydrate (24 h), C5b-8–treated cells (24 h), C5b-9–treated cells (24 h), and C5b-9–treated cells with 200 μg/ml Dynasore hydrate (24 h). Upon completion of the treatments, cells growing on culture well inserts were fixed with 2% w/v paraformaldehyde/2% w/v glutaraldehyde for 2 h before incubating them in 1.5% w/v osmium tetroxide/1.5% w/v potassium ferricyanide for 1 h. Cells were dehydrated using increasing concentrations of ethanol (70, 90, and 100% v/v ethanol), followed by propylene oxide before embedding in EPON resin. The insert membrane was cut into 70-nm sections using a Leica UC7 ultra-microtome and imaged on a JEOL 1010 transmission electron microscope. Mitochondria counting in RPE cells was performed using ImageJ. The number of mitochondria counted was normalized to the cytoplasmic area/cell, excluding the area covered by the nucleus.

## Results

### Transient assembly of C5b-9 on RPE cells

**FIGURE 1.**
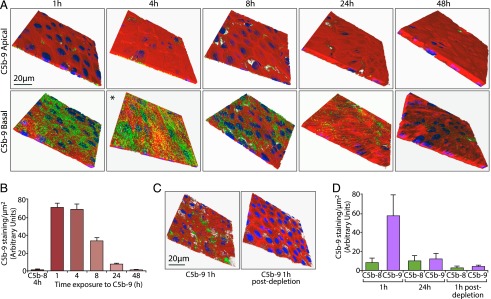
Accumulation and elimination of C5b-9 on the basal RPE cell surface. (**A**) Porcine RPE cells were cultured on Transwells and incubated for the periods indicated with DMEM in the apical chamber, as well as with DMEM containing C5b-6, C7, C8, and C9 in the basal chamber. C5b-9 (green), F-actin (red), and DAPI (blue) were visualized by confocal microscopy in full-thickness Z-stacks, of which the apical and basal cell surfaces are shown. *A 3D view is provided in Supplemental Video 1. (**B**) Quantitation of C5b-9 staining is presented as the average fluorescence intensity of 12 full-depth fields of view from three independent experiments. Omission of C9 from the complement protein mix provided the control C5b-8 sample, which was used in this experiment and elsewhere in this study to demonstrate the specificity of the Ab for C5b-9. (**C**) RPE cell monolayers were incubated for 1 h as in (A) (*left panel*) or with complement-containing medium that had been in contact with RPE cells for 24 h (*right panel*). Monolayers were processed for imaging as in (A). (**D**) RPE cell monolayers were treated as in (A) for 1 and 24 h, after which the basal DMEM containing the complement protein mix was applied to a new Transwell for 1 h to generate the 1-h postdepletion data. The results show that basal medium taken after 24 h exposure to RPE cells does not support de novo C5b-9 formation. Data were generated from four separate fields of view from three independent experiments and are expressed as mean ± SEM.

To investigate the effects of C5b-9 on RPE cells, we first established an experimental model in which primary porcine RPE cells were cultured on Transwells, and purified complement proteins were added to the basal compartment at concentrations corresponding to those found in normal human serum. C5 is synthesized primarily in the liver, and its concentration in human plasma (derived from normal donors) ranges from 70 to 170 μg/ml (38–40). Therefore, to mimic the in vivo concentration of C5 protein in the circulation, C5b-6 was used at 150 μg/ml. The use of C5b-6 obviated cleavage of C5 protein into C5a and C5b (41). C7 is mainly produced by the liver and bone marrow, and its concentration varies from 50 to 70 μg/ml in human serum (42, 43). C8 is composed of three subunits (α, β, and γ), and its concentration in human plasma varies between 50 and 80 μg/ml (44, 45). C9 is the final component of the C5b-9 complex, and its concentration in human serum ranges from 40 to 70 μg/ml (42, 46). The complement proteins were added in serum-free medium rather than whole serum because the presence of growth factors and other bioactive molecules in serum could obscure the specific effects of C5b-9. Cultures were fixed and immunostained for C5b-9 and F-actin, and the full thickness of the monolayer was rendered in 3D by confocal microscopy (Fig. 1A, Supplemental Video 1). Within 1 h of exposure to the mix of complement proteins, there was abundant punctate staining for C5b-9 on the basal RPE cell surface, but none was detected on the apical surface, demonstrating both the integrity of the monolayer and the absence of basal-to-apical transcytosis of the intact complex. Quantification of total cellular C5b-9 staining showed that, by 8 h, the levels of C5b-9 had decreased by ∼50%; by 24 h, the complex had almost completely disappeared (Fig. 1B).

In these experiments and elsewhere in this study, we used a control in which C9 was omitted to demonstrate the specificity of the C5b-9 Ab (which only recognizes C9 in the complex), as well as to show that effects on RPE cells were specific to C5b-9. Thus, if C5b-8 is indicated (Fig. 1B, 1D), this refers to the staining obtained using the C5b-9 Ab when C9 was not included in the complement protein mix. We considered three testable hypotheses to explain the decline in C5b-9 staining. The first was depletion of complement proteins from the media with concomitant elimination of C5b-9 by the RPE cells; the second was an acquired resistance to C5b-9 by the cells (e.g., through upregulation of negative complement regulators, such as CD59); and the third was C5b-9 complex formation in solution that could not become cell associated. To test the first hypothesis, basal media containing the C5b-9 complement components were conditioned by exposure to RPE cells for 24 h, and these (or fresh media) were applied to new monolayers for 1 h. The results show that the 24-h conditioned media failed to support C5b-9 formation, suggesting that the complement proteins were indeed depleted from the media under these experimental conditions (Fig. 1C, 1D). Western blot analysis failed to reveal any change in the expression of DAF/CD55 or CD59 during the 48-h exposure to C5b-9 (data not shown). To test the third hypothesis, the complement protein mix was incubated for 24 h prior to addition to the cells, but this did not lead to any difference in C5b-9 staining compared with adding unmixed proteins (Supplemental Fig. 1A). This observation does not rule out the formation of soluble C5b-9 complexes that then become cell associated by a potential RPE cell–derived complement activator, although secretion of vitronectin by RPE cells may counteract such a mechanism (32).

### RPE cells eliminate C5b-9 via the endocytic pathway

By using the purified proteins we achieved C5b-9 activation without the need for any upstream complement activation, a technique known as reactive lysis (47). However, this approach can lead to peripheral binding of the C5b-9 complex without full insertion into the plasma membrane. To address this possibility, we assembled C5b-9 as described earlier and then briefly exposed the cells to trypsin, because fully inserted or internalized C5b-9 would be expected to be resistant (Supplemental Fig. 1B). Although there was a reduction in the amount of C5b-9 staining in the trypsin-treated samples, this was not significant, and the confocal images revealed abundant staining of C5b-9 associated with the cells. Therefore, we sought to determine the mechanism responsible for the clearance of C5b-9 by RPE cells. It was shown that other cell types, such as oligodendrocytes, platelets, and tumor cells, protect themselves from C5b-9–induced lysis by membrane vesiculation (48), but the route of C5b-9 elimination in RPE cells is not known.

**FIGURE 2.**
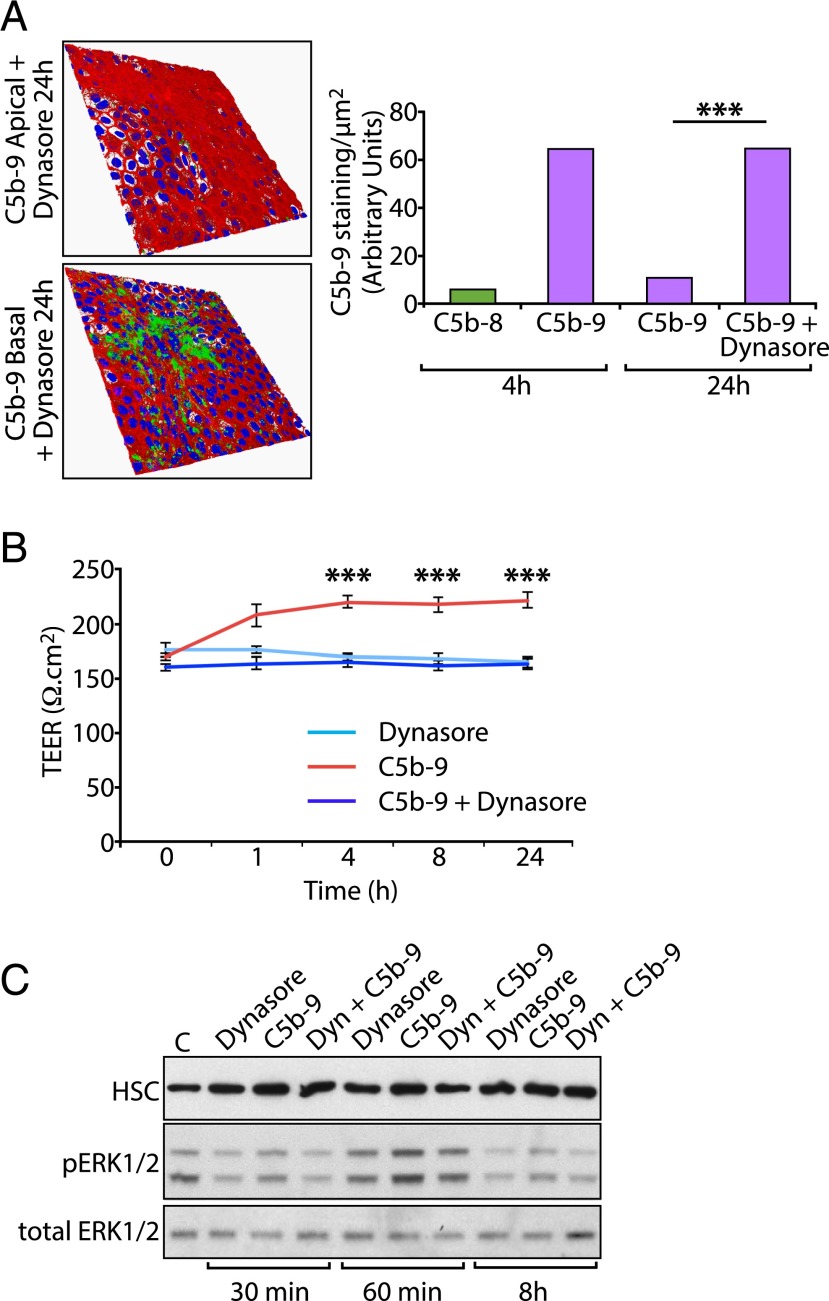
Endocytosis is required for the elimination of surface-associated C5b-9. (**A**) RPE cell monolayers were cultured on Transwells for 24 h in the presence or absence of 200 μM Dynasore, with DMEM containing the complement protein mix in the basal chamber. Cells were processed for imaging as in Fig. 1A. Quantitative analysis shows the expected accumulation of C5b-9 at 4 h and subsequent decline at 24 h. However, in the presence of Dynasore, the level of C5b-9 staining at 24 h was significantly higher and had not decreased significantly from the value at 4 h. (**B**) To assess the integrity of the monolayer during prolonged exposure to C5b-9 and Dynasore, TEER was measured during the 24-h experimental period. C5b-9 elicited a small, but significant, increase in TEER during this period that was abolished in the presence of Dynasore. Dynasore alone had no effect on TEER. Data are expressed as mean ± SEM [*n* = 12 fields of view from three independent experiments (A) or 12 wells/condition (B)]. (**C**) To examine ERK1/2 activation, RPE cells were incubated with 200 μg/ml of Dynasore alone, C5b-9 alone, or the two in combination. Cells were extracted at the times indicated, and whole-cell lysates were analyzed by SDS-PAGE and Western blotting using Abs against p-ERK1/2, total ERK1/2, and heat shock protein 70-kDa chaperone as the loading control. Protein bands were visualized by ECL. ***p < 0.001.

To investigate the possible involvement of the endocytic pathway we used Dynasore, a noncompetitive inhibitor of the GTPase activity of dynamins I, II, and III that blocks both clathrin- and caveolae-mediated endocytosis (49, 50). We observed that, in RPE cells exposed to Dynasore, C5b-9 was almost completely retained at the cell surface 24 h after incubation with the mix of complement proteins (Fig. 2A), suggesting that the major mechanism for removal of C5b-9 from the cell surface is endocytosis. During our experiments we recorded TEER to monitor the integrity of the monolayer and observed that C5b-9 elicited a small, but reproducible, increase in TEER that was abolished in the presence of Dynasore (Fig. 2B). Thus, resting TEER ∼ 150 Ω/cm^2^ was increased to >200 Ω/cm^2^ in the presence of C5b-9, with no effect observed with Dynasore alone. Interestingly, elevated TEER upon exposure to C5b-9 was sustained up to 24 h, by which time C5b-9 is almost completely absent from the basal RPE cell surface (Fig. 1B). These results suggest that the dynamin-dependent intracellular trafficking, and not the presence of C5b-9 on the cell surface per se, drives the change in TEER. The reasons for this are not clear, and examination of certain major junctional proteins, such as claudin-19 and ZO-1, did not reveal any changes in immunolocalization that might explain this observation (results not shown). However, we observed transient activation of ERK1/2 in response to C5b-9, consistent with previous investigations in RPE cells (33), which was reduced in the presence of Dynasore and occurred with similar kinetics to the initial increase in TEER (Fig. 2C).

### Intracellular trafficking of C5b-9 in RPE cells

**FIGURE 3.**
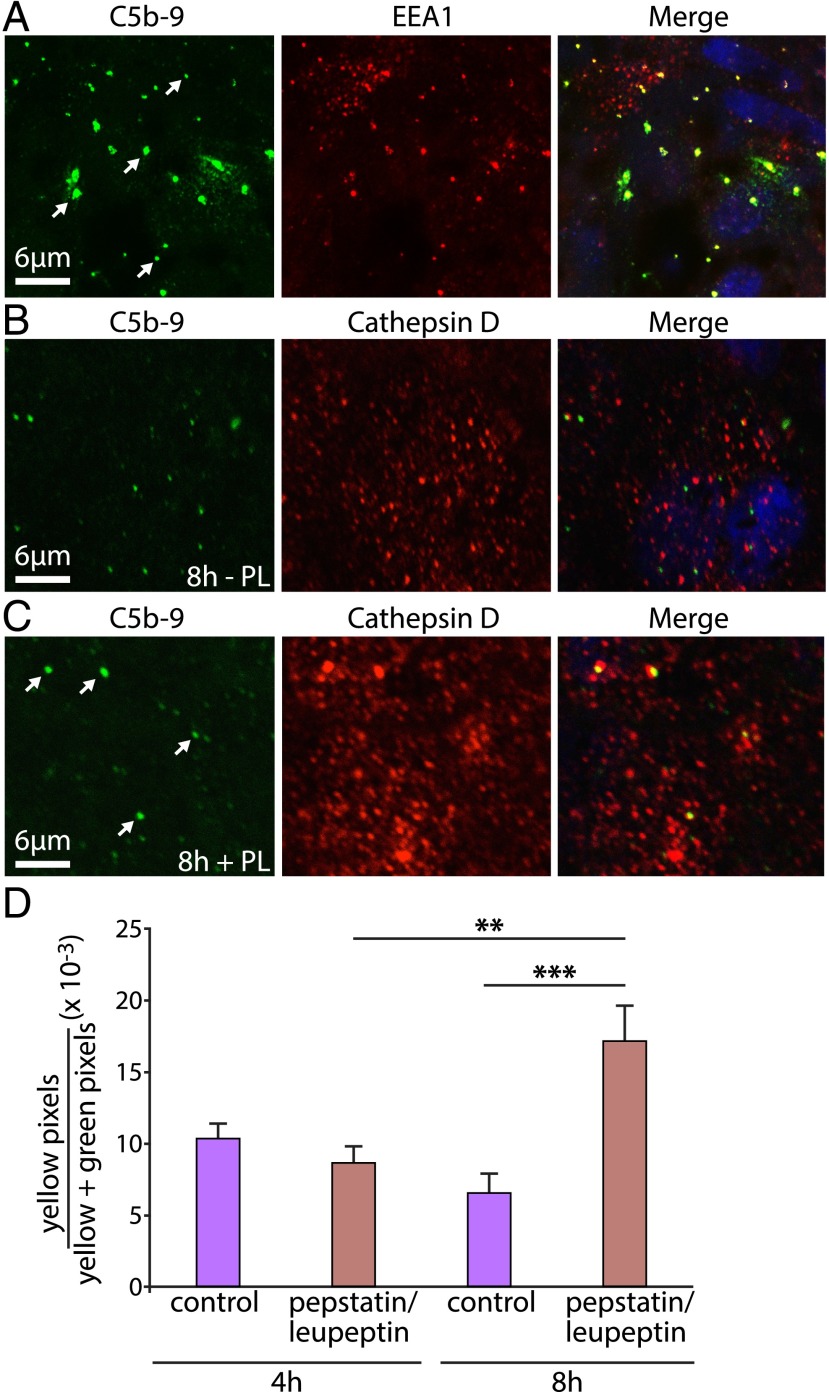
Clearance of C5b-9 via the endocytic pathway. (**A**) RPE cell monolayers were cultured on Transwells for 4 h in the presence of DMEM containing the complement protein mix and then fixed and immunostained for C5b-9 (green), the early endosomal marker EEA1 (red), and nuclei (DAPI; blue). The representative images show puncta of C5b-9 (white arrows), many of which colocalize with EEA1. (**B**) Cells were treated as above for 8 h and then fixed and immunostained for C5b-9 (green) and the lysosomal marker cathepsin D (red). Under control conditions (-PL) we observed little colocalization of the two markers. (**C**) Using the same conditions as in (B) but maintaining the cells in the presence of pepstatin A and leupeptin (+PL), we observed partial colocalization of C5b-9 (white arrows) with cathepsin D, consistent with its trafficking to the lysosome. Note that the variability in DAPI staining is due to the position of the confocal slice relative to the nucleus. (**D**) Bar graph shows the relative proportion of C5b-9 staining that colocalized with cathepsin D at 4 and 8 h, in the presence and absence of pepstatin A and leupeptin. The numbers were calculated by counting the yellow pixels (corresponding to C5b-9 and cathepsin D colocalization) and dividing these values by the number of yellow plus green (C5b-9) pixels. Data are expressed as mean ± SEM (*n* = 5 images/experiment from three independent experiments). In the presence of the protease inhibitors there was a significant (**p < 0.05) increase in the colocalization of C5b-9 and cathepsin D from 4 to 8 h and likewise when comparing the control sample at 8 h with the cells treated with the inhibitors (***p < 0.001).

Retention of C5b-9 at the basal cell surface following Dynasore treatment suggested that, under normal conditions, the majority of C5b-9 is removed from the surface of RPE cells via endocytosis. To further investigate the postendocytic fate of C5b-9 from the basal RPE cell surface, we immunostained monolayers for C5b-9 and the early endosomal marker EEA1. We observed that C5b-9 staining appeared as irregular-shaped puncta and that many of the C5b-9^+^ structures colocalized with EEA1 (Fig. 3A). When we performed the same experiment using whole human serum instead of purified complement proteins, we also observed colocalization of C5b-9 with EEA1, but with clustering of the early endosomes presumably induced by other serum factors (Supplemental Fig. 2). The extensive colocalization of C5b-9 with EEA1 after 4 h of incubation, coupled with reduced cell-associated C5b-9 after 8 h of incubation, suggests that the endocytosed complex might be delivered to the lysosome for degradation. However, after 4 and 8 h of incubation with C5b-9 in control conditions, there was only occasional colocalization of C5b-9 with the lysosomal hydrolase cathepsin D (Fig. 3B). EGF-stimulated EGFR, which is also endocytosed into EEA1^+^ early endosomes and then delivered to the lysosome for degradation, shows little costaining with lysosomal markers unless the cells are incubated with lysosomal enzyme inhibitors to prevent receptor degradation (51). To determine whether the low incidence of colocalization of C5b-9 was also due to rapid degradation of the Ab epitope on lysosomal delivery, we tested the effects of incubation with a combination of the protease inhibitors pepstatin A and leupeptin on colocalization of endocytosed C5b-9 with cathepsin D. Under these conditions, from 4 to 8 h, we observed a significant increase in the number of lysosomes that were positive for C5b-9, consistent with an accumulation of C5b-9 in the lysosomal compartment in the presence of the protease inhibitors (Fig. 3C, 3D). Taken together, these observations demonstrate that RPE cells eliminate C5b-9 via the endocytic pathway and lysosomal degradation.

### Retention of C5b-9 at the cell surface leads to mitochondrial abnormalities

**FIGURE 4.**
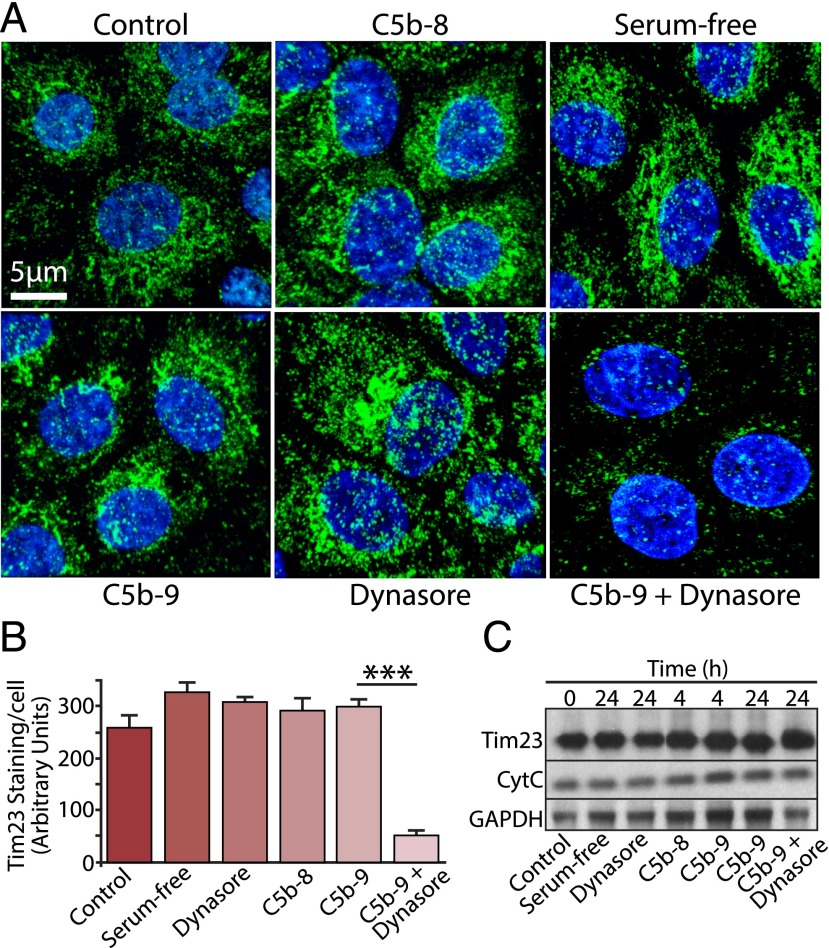
Persistent exposure to C5b-9 leads to mitochondrial perturbation. (**A**) RPE cells were cultured on Transwells for 24 h and exposed to a variety of experimental conditions, as indicated in the figure. Cells were fixed and immunostained for the mitochondrial marker Tim23 (green) and DAPI (blue). (**B**) Data are expressed as mean ± SEM (*n* = 4 images/experiment from three independent experiments). Quantitative analysis of Tim23 staining revealed a significant reduction (***p < 0.001) in staining intensity in cells treated with C5b-9 and Dynasore versus C5b-9 or Dynasore alone. (**C**) Whole-cell lysates were prepared from RPE cells cultured under the same set of conditions as above for the times indicated and Western blotted for Tim23, cytochrome C (CytC), and GAPDH as a control. No discernable difference was noted in the band intensities for either mitochondrial protein, under any of the different conditions.

**FIGURE 5.**
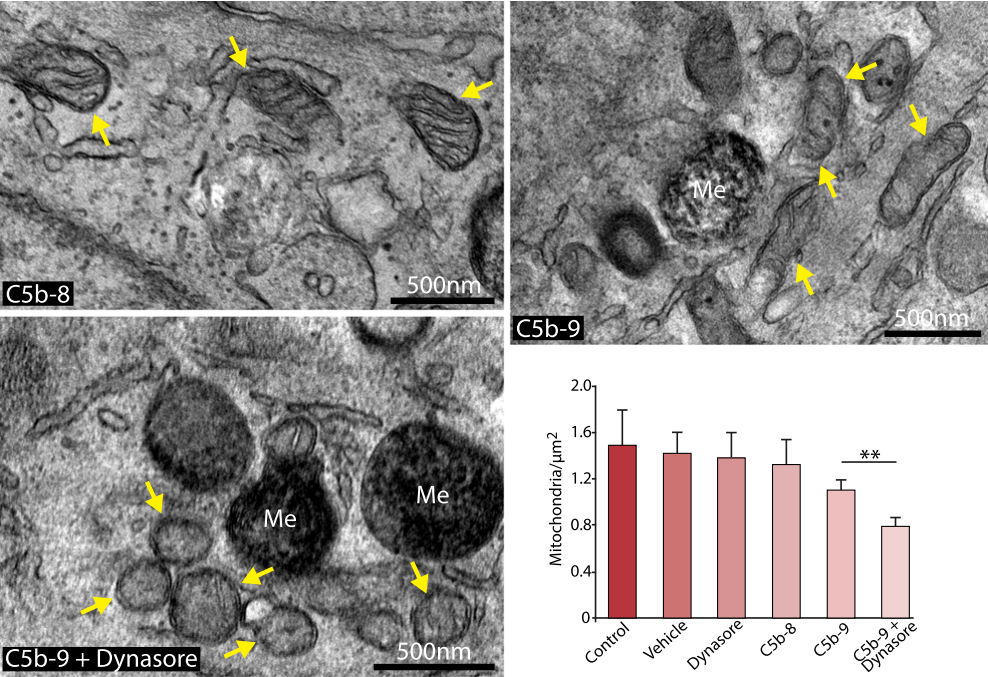
Ultrastructural defects in mitochondria exposed to persistent C5b-9. The images show representative transmission electron micrographs of RPE cells that were cultured in Transwells in the presence of C5b-8 (control), C5b-9, and C5b-9+Dynasore for 24 h. Mitochondria are highlighted by arrows. The images show that, in the presence of C5b-9+Dynasore, mitochondria are smaller and rounder than in the other conditions. The bar graph shows a quantitative enumeration of mitochondria number as a function of area, with cells treated with C5b-9+Dynasore exhibiting significantly fewer mitochondria than cells treated with C5b-9 alone. Data are expressed as mean ± SEM (*n* = 5 images/experiment from three independent experiments). **p < 0.05. Me, melanosome.

Having demonstrated that C5b-9 is normally removed from the basal RPE cells via the endocytic pathway with no apparent adverse effects on the cells, we then asked how the cells would respond if this process was perturbed. Because mitochondrial dysfunction may contribute to loss of RPE cell viability in AMD (36, 52), we evaluated the expression of mitochondrial markers in cells exposed to Dynasore and C5b-9 for 24 h. Immunofluorescence analysis revealed that blocking the endocytosis of C5b-9 led to a significant reduction in staining intensity of the mitochondrial membrane protein Tim23 (Fig. 4A, 4B) that was not observed in cells treated with Dynasore alone, C5b-8, or C5b-9. Similar observations were obtained using cells loaded with MitoTracker (Supplemental Fig. 3). However, Western blotting of whole-cell lysates revealed that, despite the loss of mitochondrial staining, total cellular levels of both Tim23 and cytochrome C were unchanged (Fig. 4C), showing that the apparent loss of Tim23 staining was due to failure to concentrate the protein in mitochondria, rather than a reduction in gene expression. We then used electron microscopy to examine mitochondrial ultrastructure; consistent with the results in Fig. 4, we observed that there were significantly fewer mitochondria in RPE cells treated with Dynasore and C5b-9 than in cells treated with C5b-9, C5b-8, vehicle, or Dynasore alone (Fig. 5, Supplemental Fig. 4). Furthermore, the mitochondria in the cells treated with Dynasore and C5b-9 tended to be smaller, rounder, and had fewer discernable cristae. These observations suggest that if C5b-9 persists at the cell surface, it may lead to changes both in mitochondrial morphogenesis and the recruitment and targeting of mitochondrial proteins that would be expected to have deleterious consequences on aspects of RPE cell function, such as energy production and Ca^2+^ handling.

## Discussion

RPE cells form the posterior blood–retinal barrier in the eye, where, in association with the underlying Bruch’s membrane, it comes into direct contact with the systemic pool of circulating complement proteins. Numerous reports showed that, in AMD, individual complement proteins, as well as the C5b-9 complex, become enriched in drusen and in the basal RPE cell/Bruch’s membrane, and experimental models using cultured RPE cells demonstrated that C5b-9 has the potential to modulate RPE cell function. However, it is also clear that RPE cells, like many other cells, have molecular mechanisms and regulatory proteins that enable them to largely evade the potentially harmful effects of complement activation. The complement regulators include cell surface proteins, such as CD59, although our observation that C5b-9 forms readily on RPE cells in the absence of CD59 blocking Abs, suggests that the level of expression of CD59 is insufficient to regulate C5b-9 formation in this experimental model. In AMD, it was reported that levels of complement regulators, such as CD59, are reduced in disease-affected areas (53), which would be expected to render the RPE cells vulnerable to attack by C5b-9. In this study, we sought to elucidate the mechanism(s) used by RPE cells to eliminate C5b-9, because deficits in this process may contribute to the RPE cell dysfunction associated with AMD pathogenesis. First, we established a cell culture model for C5b-9 assembly that used primary RPE cells in confluent monolayers on culture well inserts. In all studies we verified the integrity of the monolayers by measuring TEER, and we aimed to conserve RPE cell properties by restricting the cells to only a few rounds of division prior to experimentation. Using purified complement proteins in serum-free media, we observed that C5b-9 formed rapidly on the basal RPE cell surface, as judged by immunofluorescence analysis, which was cleared over 24–48 h with no apparent detrimental effects to the cells. A number of studies reported that nucleated cells use various strategies to eliminate surface-associated C5b-9, including endocytosis, ectocytosis, and exocytosis (54–56), each of which may be used in a cell type–specific manner, and all have been postulated as possible mechanisms for C5b-9 removal in RPE cells (57). However, no previous studies directly addressed this question in RPE cells, and those studies that reported that C5b-9 is removed by endocytosis did not show whether the fate of the internalized complex is lysosomal degradation. In this study, we observed that the dynamin inhibitor Dynasore completely blocked the removal of C5b-9 from the basal RPE cell surface. These results suggest that, in RPE cells, C5b-9 may be processed in a similar manner to that reported in K562 erythroleukemia cells, where C5b-9 was shown to colocalize with caveolin-1, and internalization was dependent on dynamin-2 (56).

One striking and highly reproducible finding was that the C5b-9 complex increases TEER in RPE cells. Little is known about the relationship between exposure to C5b-9 and RPE cell barrier function; however, two studies reported no change in TEER upon formation of sublytic C5b-9 on RPE cells (58, 59), whereas another reported a decrease in TEER, although this required additional oxidative stress (33). These contrasting observations are probably due to the use of different experimental models, because both previous studies used ARPE19 cells, and C5b-9 assembly was performed at the apical cell surface using whole serum as a source of complement proteins. Moreover, in the latter study, the loss of TEER was shown to be a secondary effect, due to an increase in expression and secretion of vascular endothelial growth factor. The increase in TEER observed in this study is somewhat unexpected because pathogenic stimuli are normally associated with a decrease in barrier function. However, sublytic C5b-9 was shown to activate ERK1 and RhoA in various cell types (60, 61), consistent with our observation of ERK1/2 activation by C5b-9 in this study, and RhoA activation was shown to tighten epithelial junctions in kidney epithelial cells (62). We also observed that TEER was elevated when C5b-9 was assembled on the apical RPE cell surface (data not shown), further supporting the idea that intracellular trafficking of the complex appears to stimulate the increase in TEER rather than its presence at the cell surface.

The ability of normal healthy RPE cells to withstand the potentially harmful effects of exposure to C5b-9 is consistent with observations in other nucleated cell types; however, in AMD, chronic exposure to C5b-9 may be linked to oxidative stress and inflammatory RPE cell responses. We asked whether inhibiting the internalization and degradation of C5b-9 by blocking the endocytic pathway would specifically elicit effects on mitochondria, because mitochondrial dysfunction is closely associated with oxidative stress. Using immunofluorescence analysis of MitoTracker and Tim23, together with electron microscopy to obtain ultrastructural information, we observed a reduction in the number and size of mitochondria, damage to cristae, and the loss of internal mitochondrial membranes. Despite these alterations the RPE cells remained viable, with maintenance of TEER and no evidence of apoptosis, as judged by TUNEL staining (data not shown). Similar changes in mitochondrial morphology were reported in skeletal muscle from mice lacking p53, in which a marked reduction in Tim23 delivery to mitochondria was also observed (63), as well as in RPE cells from AMD patients (64). Interestingly, proteomic analysis of human RPE cells isolated from AMD patients revealed that aged RPE cells were characterized by alterations in their mitochondrial protein content compared with control samples (65). Mitochondrial rounding is more commonly associated with failures in cellular Ca^2+^ handling, as reported in models of amyotrophic lateral sclerosis (66) and endothelial cells (67). The link with Ca^2+^ signaling may be particularly relevant given that, in cultured RPE cells, C5b-9 was shown to elicit a transient increase in Ca^2+^ that, under certain conditions, can lead to cell death (27, 28, 33, 68).

In summary, we showed that RPE cells dispose of surface-bound C5b-9 via the endocytic pathway and lysosomal degradation and that this is important because failure to eliminate C5b-9 leads to changes in mitochondrial morphology that could compromise cellular activities. In aging, it was shown that lysosomal capacity in the RPE cells decreases, possibly due to the accumulation of lipofuscin. Thus, blue light irradiation of lipofuscin-loaded human RPE cells and ARPE-19 cells was shown to cause photo-oxidative damage, lysosomal membrane permeabilization, and leakage of lysosomal enzymes into the cytosol (69). This cellular response induced NLRP3 inflammasome activation via upregulation of caspase-1, IL-1β, and IL-18. NLRP3 was suggested to have the capacity to impair both autophagy (removal of damaged organelles and proteins) and photoreceptor outer segment phagocytosis in RPE cells (70, 71). In addition, lipofuscin accumulation can impair autophagy (which can overlap with the endocytic pathway) by preventing lysosomal enzymes from degrading functional lysosomes (72, 73). Advanced accumulation of the nondegradable lipofuscin in the lysosomes eventually compromises the lysosomal system and, therefore, increase cellular levels of reactive oxygen species in RPE cells (74). Our observations raise the possibility that, in AMD, elevated levels of lipofuscin could impede the processing and degradation of C5b-9, creating a vicious cycle that, in turn, renders RPE cells more susceptible to complement attack.

## Supplementary Material

Data Supplement
