# Supplementary material for: Retinal Pigment Epithelial Cells Mitigate the Effects of Complement Attack by Endocytosis of C5b-9
Source: J Immunol. 2015 Aug 31;195(7):3382–9. doi: 10.4049/jimmunol.1500937 (PMC4574521; doi:10.4049/jimmunol.1500937)
Supplement: Data Supplement [file JI_1500937.zip › JI_1500937_Supplemental_Material_1.pdf]

Retinal Pigment Epithelial Cells Mitigate the Effects of Complement Attack by Endocytosis of C5b-9

Supplemental Information

Movie S1

The movie shows the full thickness 3D-rendered RPE monolayer used to generate the ‘4h C5b-9 basal’ image panel in Fig. 1A. In the movie F-actin is stained red, nuclei are blue, and C5b-9 is green. For orientation purposes, the basal cell surface is highly enriched in C5b-9.

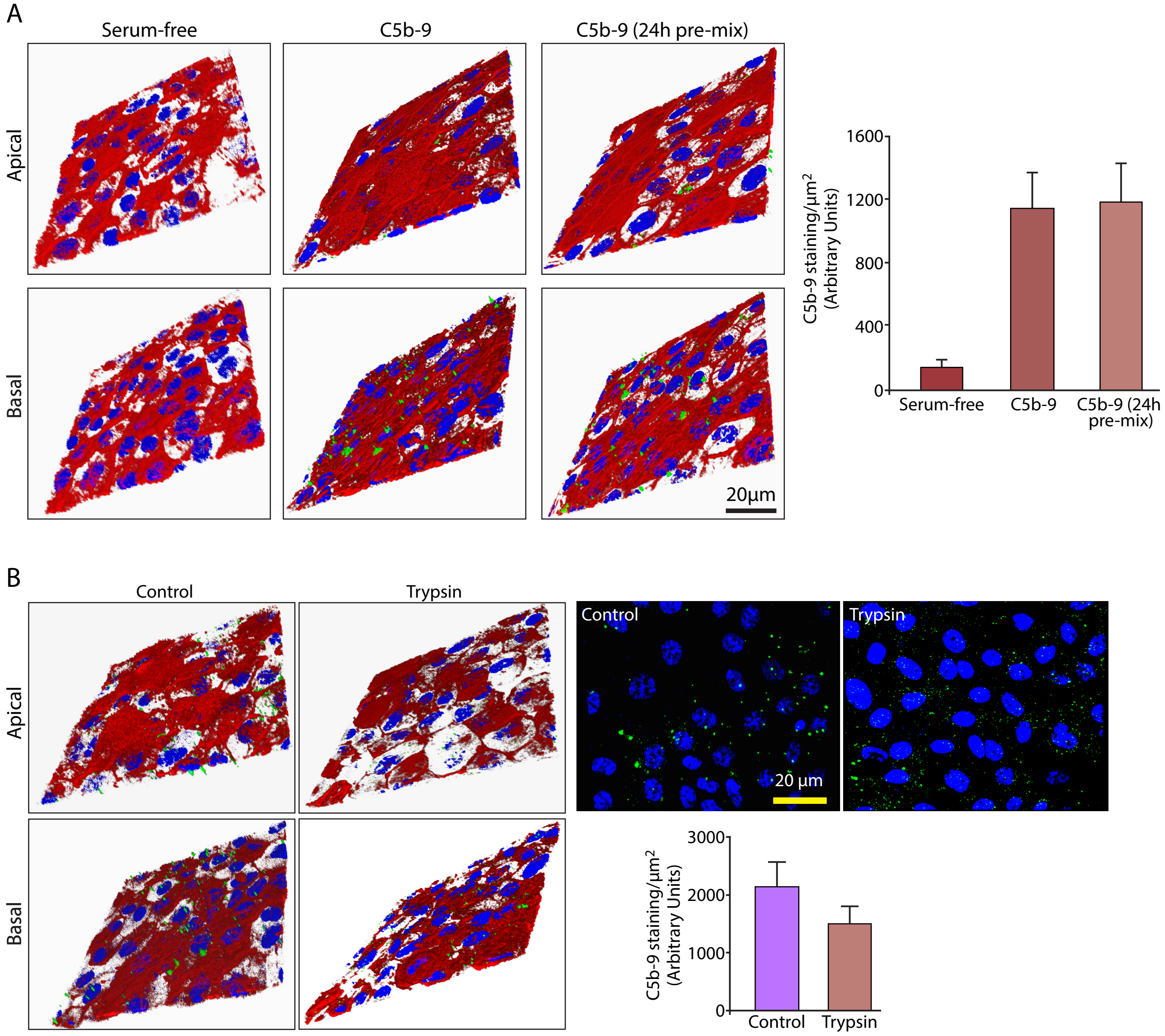

Figure S1. (**A**) Pre-mixing complement proteins does not prevent C5b-9 assembly. Porcine RPE cells were cultured on transwells and the basal surfaces were exposed for 1h to DMEM containing C5b-6, C7, C8 and C9 proteins that were either freshly added, or that had been pre-mixed in DMEM for 24h at 37ºC. The apical cell surfaces were exposed only to DMEM. C5b-9 complex formation (green) was detected via immunofluorescence using an anti-C5b-9 monoclonal antibody. The apical cell surface did not exhibit any C5b-9-positive staining whereas quantitative analysis revealed that the levels of C5b-9 complex formation on the basal surface were similar regardless of whether the complement proteins had been pre-incubated in DMEM for 24h. Red staining corresponds to F-actin, blue staining to DAPI. n = 3, with 3 fields counted per experiment. (**B**) Trypsinisation of surface-bound C5b-9 on RPE cells. Porcine RPE cells were cultured on transwells and the basal surfaces were exposed to DMEM containing C5b-6, C7, C8 and C9 proteins for 4h at 37ºC. The apical cell surfaces were exposed only to DMEM. Prior to fixation, the basal chambers were incubated with PBS (control) or with PBS containing 1 % Trypsin for 5 min at 37ºC. Cells were then fixed and stained for C5b-9 (green staining) using an anti-C5b-9 mouse monoclonal antibody. The en face images and histogram show that trypsinisation reduced the amount of C5b-9 staining, though the difference was not significant. Red staining corresponds to F-actin, blue staining to DAPI. n = 3, with 3 fields counted per experiment.

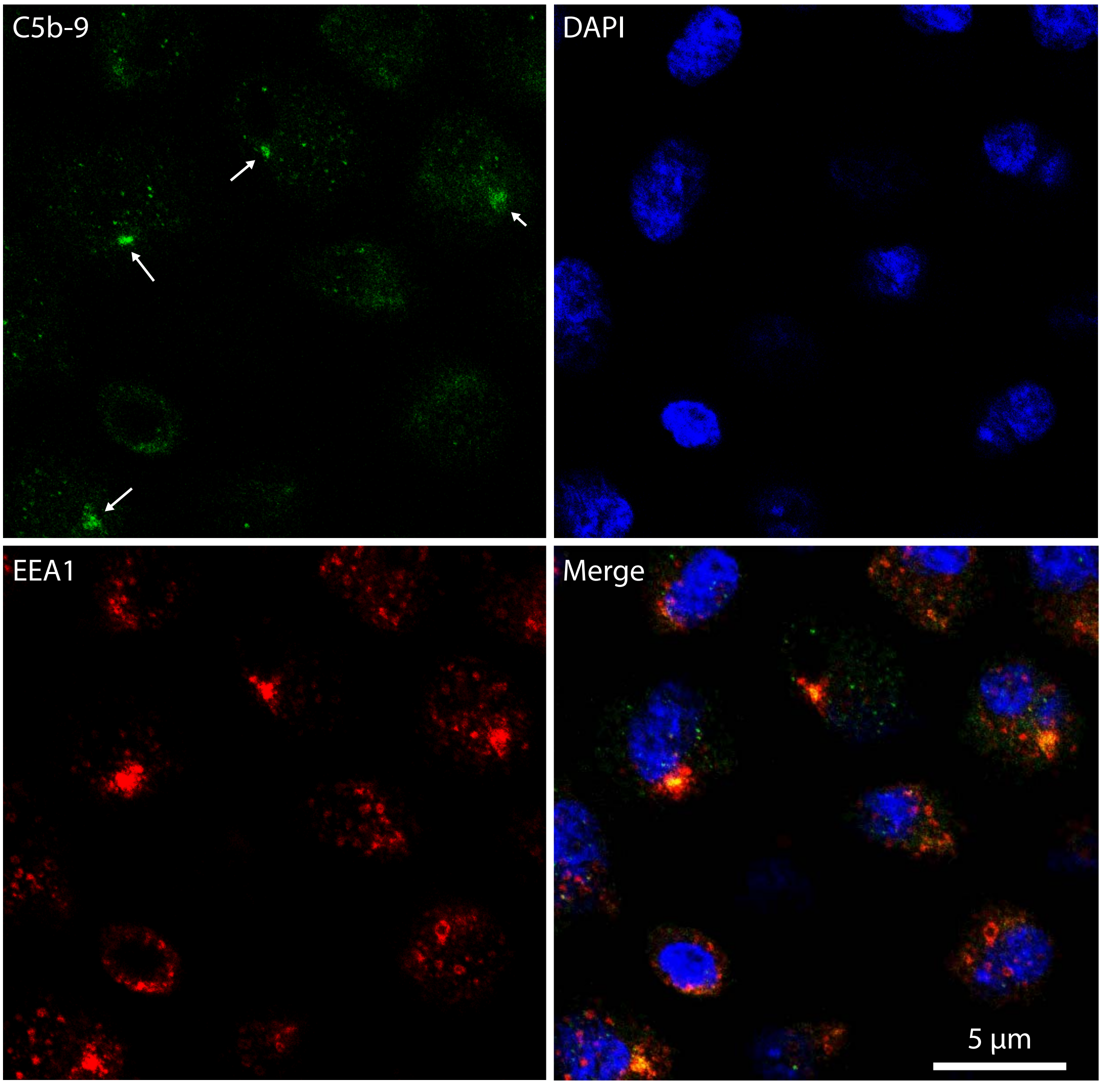

Figure S2. Normal human serum induces C5b-9 formation on pRPE cells. Porcine RPE cells were cultured on transwells and the basal surfaces were exposed to either 100 % heat-inactivated or non-heat-inactivated normal human serum (NHS) for 4 h. Cells were fixed and stained for C5b-9 (green) and the early endocytosis marker EEA-1 (red). No C5b-9 staining was observed using heat-inactivated NHS (not shown), but non-heat-inactivated NHS supported C5b-9 formation, which by 4 h was also partially co-localised with EEA1. Blue staining corresponds to DAPI. Scale bar represents approximately 6µm.

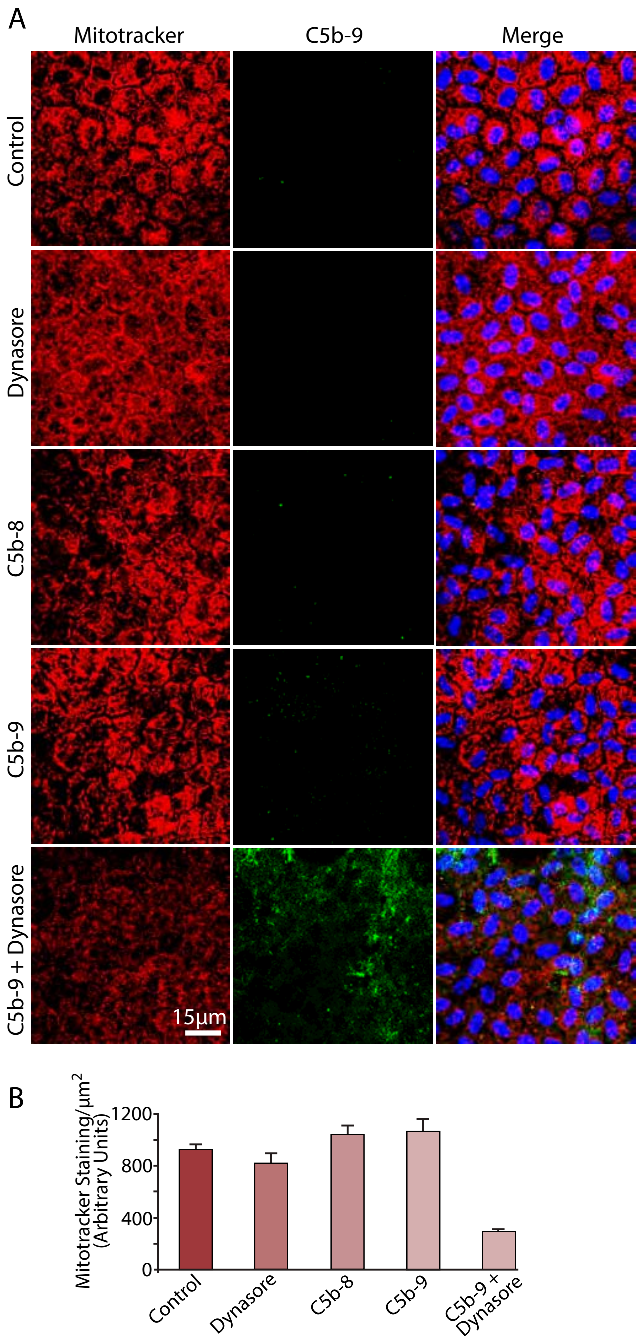

Figure S3. Persistent exposure to C5b-9 leads to mitochondrial perturbation. (**A**) RPE cells were cultured on transwells for 24h and a variety of experimental conditions were applied to the basal chamber as indicated in the figure. Mitotracker Red CMXRos (red staining) was added to the apical chamber of all samples 30 minutes before the cells were fixed and immunostained for C5b-9 (green) and DAPI (blue). (**B**) Quantitative analysis of Mitotracker staining revealed a reduction in staining intensity in cells treated with C5b-9 and Dynasore, when compared to C5b-9 or Dynasore alone. Data are expressed as mean ± SEM, n = 3 images per sample from one experiment.

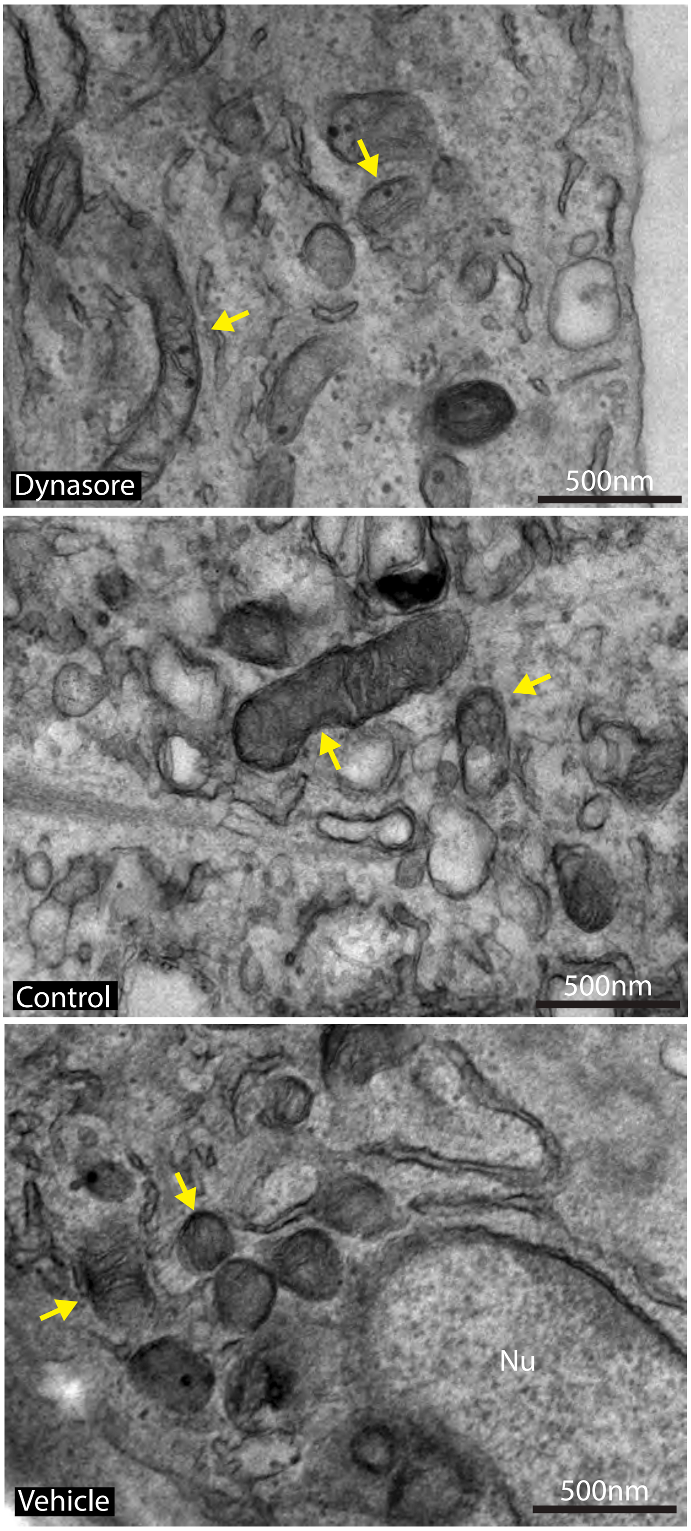

Figure S4. Ultrastructural defects in mitochondria exposed to persistent C5b-9. The images show representative transmission electron micrographs of RPE cells that had been cultured in transwells in the presence of C5b-9, together with Dynasore, vehicle (DMSO) and DMEM (control) for 24h. The nucleus (Nu) is labeled in the vehicle image, and mitochondria are highlighted with yellow arrows.
